# Identification of Neuronal Cells in Sciatic Nerves of Adult Rats

**DOI:** 10.3389/fncel.2022.816814

**Published:** 2022-03-25

**Authors:** Yisheng Liu, Songlin Zhou, Lili Zhao, Xiaosong Gu

**Affiliations:** ^1^Model Animal Research Center, Nanjing University, Nanjing, China; ^2^Key Laboratory of Neuroregeneration, Ministry of Education and Jiangsu Province, Co-innovation Center of Neuroregeneration, Nantong University, Nantong, China

**Keywords:** neuronal cell bodies, transgenic rat model, crushed sciatic nerve, sciatic nerve culture, single-cell sequencing, neuronal stem-like cells

## Abstract

Prior research generally confirms that there are no neuronal cell bodies in the adult sciatic nerve. However, we occasionally find some neuronal cells in adult rat sciatic nerves, either intact or crush-injured. By whole-mount staining and optical imaging of the hyalinized sciatic nerves for *Stmn2* (a specific marker for neuronal cells), we found those neuronal cells with irregular distribution in the sciatic nerves in both crushed model and normal rats. We investigated the identity of those cells and established a cultured sciatic nerve model. Immunohistochemistry evidence both *in vivo* and *in vitro* illustrated that some of those cells are mature neurons in sciatic nerves. With single-cell sequencing of neuronal cells in adeno-associated virus (AAV)-infected sciatic nerves, we identified that some of those cells are a kind of neuronal stem-like cells. Then we constructed a *Nestin-CreERT*^2^ rat line and traced those cells with fluorescence labeling which was induced by tamoxifen. Interesting, we proved that neuronal stem-like cells could proliferate by combination of EdU incorporation with staining in the sciatic nerves of transgenic rats. Together, the discovery of neuronal cells in adult sciatic nerves will make us aware of the distribution of neurons in the peripheral nervous system. Especially our data suggest that neuronal stem-like cells could proliferate in the sciatic nerves of adult rats.

## Introduction

Substantial efforts have been devoted to exploring the components in the sciatic nerve including nerve fibers, Schwann cells, and fibroblasts. As a transient population, embryonic neural crest cells in the sciatic nerve are thought to quickly transfer from multipotent to restricted progenitors with limited capacity to self-renew before birth (Bronner and Simoes-Costa, 2016). But in fetal and adult peripheral nerves, neural crest cell derivatives give rise to multiple derivatives including neurons *in vitro* (Morrison et al., 1999; Wong et al., 2006; Gresset et al., 2015). Adult neurogenesis occurs in specific regions of the mammalian brain throughout life with critical roles in brain plasticity such as learning, memory, and mood regulation (Altman, 1962; Gage, 2019). Postnatal peripheral nervous system (PNS) neurogenesis has been discovered in mammalian parasympathetic ganglia of the head (Dyachuk et al., 2014; Espinosa-Medina et al., 2014) and gut (Uesaka et al., 2015). Adult PNS neurogenesis has been discovered in the carotid body (Pardal et al., 2007), in injured (Joseph et al., 2011; Laranjeira et al., 2011) and healthy enteric nervous systems (Kulkarni et al., 2017), and injured DRG (Gallaher et al., 2011). There compelling morphometric evidence for new born neurons in postnatal or adult rat DRG *in vivo* (Devor and Govrin-Lippmann, 1985, 1991). In a previous study, after oral BrdU administration via drinking water for 1 month after sciatic transection, only a handful of BrdU-labeled ipsilateral DRG neurons were found. It indicates that most new neurons are formed by late differentiation of a cell type that is not originally of neuronal size or morphology and did not undergo cell division (Groves et al., 2003). And there is a lack of direct evidence about the existence of neuronal cells in the sciatic nerves of adult rats.

However, confirming the *in vivo* existence of neuronal cells in sciatic nerves is challenging. In contrast to the extensive research of adult neurogenesis in the mammalian brain, we know very little about adult neuronal progenitors in the sciatic nerve (Czaja et al., 2012). Recent studies have revealed stem-like populations in DRG that displayed sphere-forming potential and multipotency *in vitro*, yet the *in vivo* presence of neuronal stem cells in DRG has not been confirmed in adult mammals (Li et al., 2007; Nagoshi et al., 2008; Vidal et al., 2015). Sensory DRG neurons are derived from the thoracolumbar region of trunk neural crest cells before birth. Cervical region neural crest cells differentiate into large-diameter neurons at first, following by the birth of small-diameter nociceptive neurons (Lawson and Biscoe, 1979). Late emigrating trunk neural crest cells give rise to boundary cap neural crest stem cells, a source of multipotent sensory-specified stem cells (Radomska and Topilko, 2017).

Many other neural crest stem cell (NCSC)-like cells in the adult mammal could give birth to neurons *in vitro*, such as in the bone marrow, cornea, heart, dental pulp, and periodontal ligament. All those cells consist of a pool of multipotency neural crest-derived cells (Aquino, 2017). However, those cells are identified with a kind of peripheral glia lineage. Moreover, some postnatal or adult cells of the peripheral glia lineage are shown to differentiate *in vivo* into enteric ganglia neurons, carotid body neurons, pigment cells, and tooth mesenchymal stromal cells (Hjerling-Leffler et al., 2005; Shakhova and Sommer, 2010; Aquino, 2017; Furlan and Adameyko, 2018). Those findings suggest that a subset of the neural crest cell population in the sciatic nerve maintain multipotency after embryonic development.

One major obstacle to study neuronal cells in sciatic nerves is a lack of methods to identify them. Many studies focus on cell isolation or *in vitro* culture of adult sciatic nerves because of the lack of a more specific *in vivo* tool (Morrison et al., 1999; Baggiolini et al., 2015). Stathmin 2 (*Stmn2* or *Scg10*) is highly expressed during neuronal stem cell development and in the damaged nerve of sensory neurons, where it is specifically expressed in neuronal cells (Mori et al., 1990; Okazaki et al., 1993). We adopted a sciatic nerve crush model in adult rats. By optical imaging of the crushed sciatic nerve tissue for Stmn2 (Shin et al., 2014), we observed neuronal cells along the adult rat sciatic nerves.

Single-cell sequencing was used to identify those neuronal cells marked by adeno-associated virus (AAV)2/9. Those cells presented a neuronal development process of neural crest cells by pseudo-time analysis. As an intermediate filament protein in neuroepithelial precursor cells, Nestin is considered a hallmark of neural stem/progenitor cells (Hockfield and McKay, 1985; Lendahl et al., 1990; Wiese et al., 2004; Dubois et al., 2006). We characterized neuronal stem cells labeled by the *Nestin-CreERT*^2^ rat line and *Nestin-Cre* rat line in the adult sciatic nerve with clonal lineage-tracing. EdU staining suggested those neuronal cells are from neuronal stem cell division but not from the direct transdifferentiating of glia cells without cell division. During embryonic development, neural crest cells migrate from the neural tube to the DRG as sensory neurons and to the sciatic nerve and dermal nerve as multipotent cells (Morrison et al., 1999; Baggiolini et al., 2015; Gresset et al., 2015). Our study revealed that neuronal cell bodies exist in the sciatic nerves of adult rats and part of those cells will proliferate in sciatic nerves.

## Results

### Sciatic Nerves of Adult Rats With Neuronal Cell Bodies Marked by Stmn2 Antibody

**FIGURE 1:**
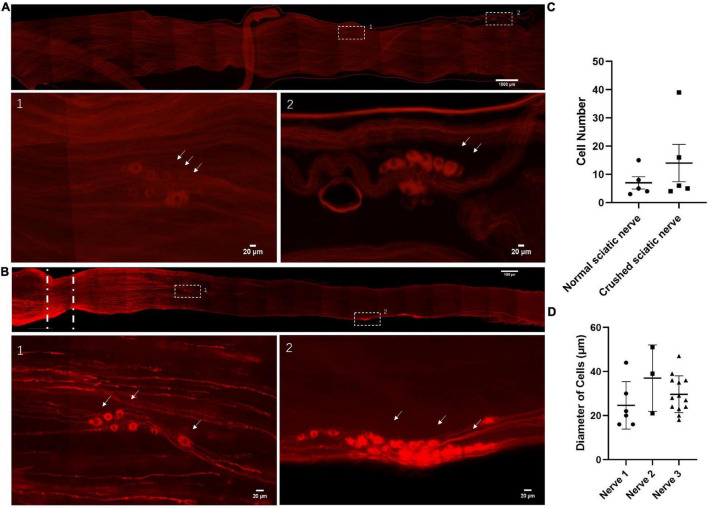
Identification of neuronal cells in the sciatic nerves of adult rats. **(A)** Neuronal cells in the intact sciatic nerves of adult rats. The nerves were hyalinized using ScaleS and stained for *Stmn2* (red). *n* = 18 (totally), *n* = 5 (sciatic nerves with neuronal cells). Scale bar: upper = 1000 μm, lower = 20 μm. **(B)** Neuronal cells in the crushed sciatic nerves of adult rats. Lower panels show high-magnification images of the boxed regions in the upper panel. The sciatic nerves were hyalinized using ScaleS and stained for *Stmn2* (red). The white lines indicated the crush site. *n* = 16 (totally), *n* = 5 (sciatic nerves with neuronal cells). Scale bar: upper = 1000 μm, lower = 20 μm. **(C)** The quantification of *Stmn2*^+^ cell number in the normal sciatic nerves and crushed sciatic nerves. The circle points represent the number of *Stmn2*^+^ cells of a normal sciatic nerve. The square points represent the number of *Stmn2*^+^ cells of a crushed sciatic nerve (Normal sciatic nerves: *n* = 5 of 18 which contain neuronal cells, crushed sciatic nerves: *n* = 5 of 16 which contain neuronal cells; *p* > 0.05; unpaired *t* test). **(D)** The quantification of diameters of *Stmn2*^+^ cells in the sciatic nerves (*n* = 3; *p* > 0.05; one-way ANOVA with Dunnett’s multiple comparisons test). The white arrow points to the positive cells.

To enable us to image deep within PNS structures, we used a clearing reagent called ScaleS that rendered the dorsal root ganglia (DRG) and sciatic nerve of adult rats transparent, but completely preserved fluorescent signals from labeled cells (Hama et al., 2015). First, we searched for the neuronal cells in adult rats and used a *Stmn2* antibody to identify those cells (Varejao et al., 2004; Wang et al., 2013; Shin et al., 2014; Gong et al., 2016). Surprisingly, we observed neuronal cell bodies along the sciatic nerves (Figure 1A). Out of 18 normal sciatic nerves, we found neuronal cell bodies in five sciatic nerves (Figure 1C). To assess whether the neuronal cells exist in damaged nerves, we used the same optical clearing method on crushed sciatic nerves of adult rats. Three days after creating a 1 mm sciatic nerve lesion, we also found neuronal cells in crushed sciatic nerves (Figure 1B). Out of 16 crushed sciatic nerves, we found neuronal cell bodies in five sciatic nerves (Figure 1C). We measured the diameters of those neuronal cell bodies (Figure 1D). Most of the neuronal cells were 25–45 μm in diameter and had two larger processes with the same morphological characteristics as DRG neurons. There was no significant difference in cell number and cell diameter between the intact or crush-injured nerves as indicated by the analysis.

### The Neuronal Cells Marked by Virus Infection *in vivo*

Delivery of AAV provides a non-invasive method for broad gene delivery to the nervous system (Foust et al., 2009). Many studies use AAVs as vehicles for gene transfer to the nervous system. Gene delivery supports gene expression and knockdown, gene editing, disease model development, *in vivo* imaging, circuit modulation, and the treatment of neurological diseases (Samulski and Muzyczka, 2014; Chan et al., 2017).

**FIGURE 2:**
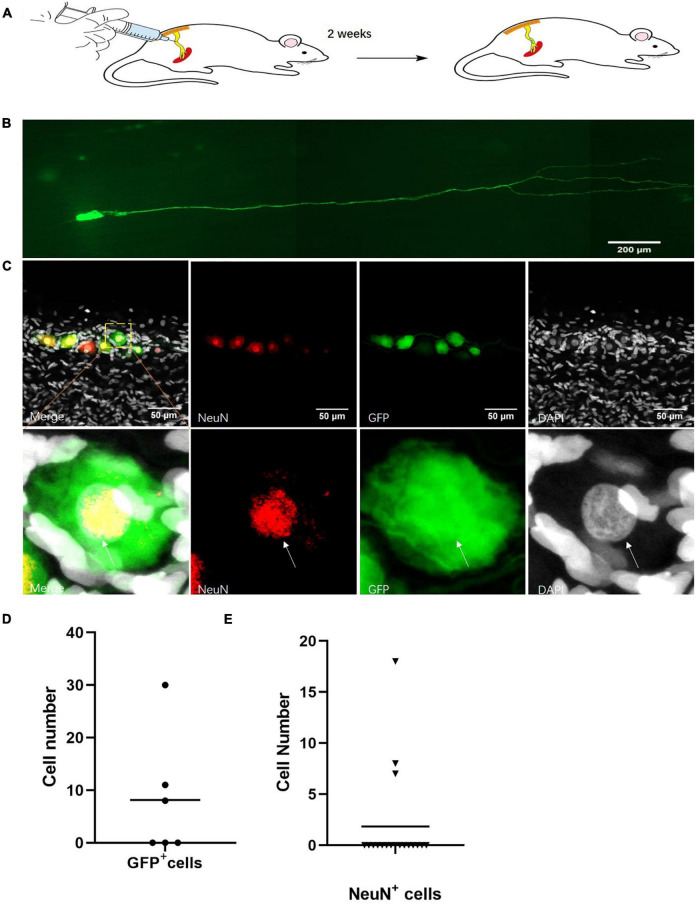
Identification of neurons in the sciatic nerves of adult rats. **(A)** Sciatic nerve infection model. *hSYN*-promoter *GFP* AAV2/9 virus was injected into the rat sciatic nerves for 2 weeks. *n* = 12. **(B)** Neuronal cells marked by *GFP in vivo*. Cells expressing *GFP* (green) in the sciatic nerves of adult rats. *n* = 6 (sciatic nerve with *GFP* marked neuronal cells). Scale bar, 200 μm. **(C)** The quantification of *GFP*^+^ cell number in sciatic nerves of virus infected rats. *hSYN*-promoter *GFP* AAV 2/9 virus was injected into the sciatic nerves. The circle points represented the cell number of *GFP*^+^ cells in the virus-infected sciatic nerve (*n* = 6 of 12 which contain the *GFP*^+^ cells). **(D)** Neurons labeled by AAV2/9 virus in sciatic nerves of adult rats *in vivo*. Two weeks after injection of *hSYN*-promoter *GFP* AAV2/9 virus, immunofluorescence staining for *NeuN* (red) and *DAPI* (gray) in *GFP* (green)-labeled cells in the sciatic nerves. *n* = 18 (total sciatic nerves with *GFP*^+^ neuronal cells), *n* = 3 (sciatic nerves with *NeuN* marked *GF*P^+^ neuronal cells). Scale bar, 50 μm. **(E)** The quantification of *NeuN*^+^ cells number in the sciatic nerve of virus injected rats. The sciatic nerves of those rats are infected by *hSYN*-promoter *GFP* AAV2/9 virus (*n* = 18). The triangular points represent the cell number of *NeuN*^+^ cells in a sciatic nerve with *GFP*^+^ cells (*n* = 3). The white arrow points to the positive cells.

To label the neuronal cells *in vivo*, we infected the sciatic nerves with engineered AAV2/9 with the *hSYN* and *hEF1a* promoter-induced fluorescent protein *GFP* (Figure 2A). We detected the neuron projections in these infected neuronal cells *in vivo* (Figure 2B). Out of 12 infected sciatic nerves, we found neuronal cell bodies in six sciatic nerves (Figure 2C). Although, only a few neuronal cells were stained in those sciatic nerves, the expression of the neuron-specific marker *NeuN* (Mullen et al., 1992) indicated a number of those cells were neurons (Figure 2D). Out of 18 sciatic nerves with neuronal cell bodies, we found *NeuN*^+^ cells in three sciatic nerves (Figure 2E).

Together, this evidence indicated that there were neurons along the sciatic nerves of adult rats.

### The Neuronal Cells Infected by Virus in Cultured Sciatic Nerves

**FIGURE 3:**
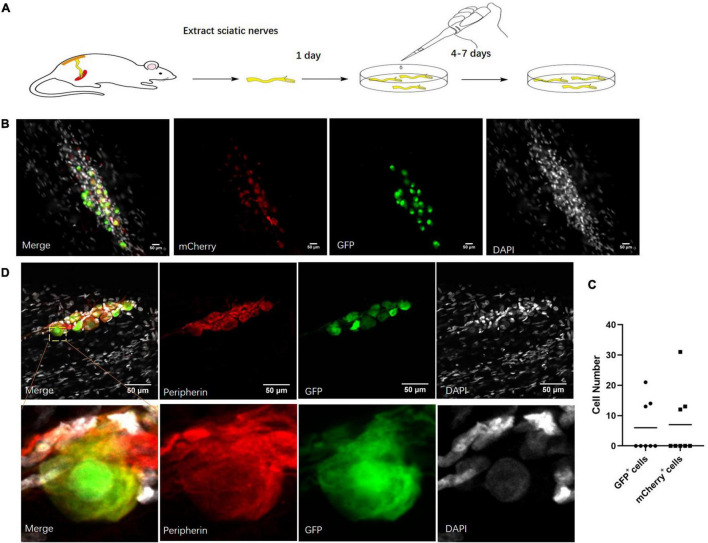
Neuronal cells marked by virus in cultured sciatic nerves. **(A)** Sciatic nerve culture model. The sciatic nerves of adult rats were cultured in a defined serum-free medium. *n* = 8 (totally). **(B)** Cultured sciatic nerves infected by *CMV*-promoter *mCherry* AAV2/9 virus and *hSYN*-promoter *GFP* AAV2/9 virus for 2 weeks. Distribution of *CMV*-promoter *GFP*^+^ cells (green) and *hSYN*-promoter *mCherry*^+^ cells (red) in the cultured sciatic nerves. *n* = 3 (sciatic nerves with *GFP*^+^ *mCherry*^+^ cells). Scale bar, 50 μm. **(C)** The quantification of *GFP*^+^ cells and *mCherry*^+^ cell number in the cultured sciatic nerves which were infected by *CMV*-promoter *mCherry* AAV2/9 virus and *hSYN*-promoter *GFP* AAV2/9 virus. The circle points represented the number of *GFP*^+^ cells. The triangular points represented the number of *mCherry*^+^ cells (*n* = 3 of 8 which contain the *GFP*^+^ *mCherry*^+^ cells). **(D)** Virus-labeled cells are neuronal cells. Immunofluorescence staining for *Peripherin* (red) and *DAPI* (gray) was performed in cultured adult rat sciatic nerves with *GFP* (green)-labeled cells after 2 weeks infection of the *hSYN*-promoter *GFP* AAV2/9 virus. *n* = 4 (all the *GFP*^+^ cells could be stained by *Peripherin*). Scale bar, 50 μm.

As neurons could only be detected in part of the sciatic nerves, the identification of other neuronal cells was very important. Because of the infrequent existence of the neuronal cells in the sciatic nerves and low efficiency of virus infection *in vivo*, we cultured the sciatic nerves in a serum-free medium. We infected the cultured sciatic nerves with AAV2/9 containing the *hSYN* or *CMV* promoter so that those cells stably expressed *GFP* or *mCherry* (Figure 3A). After more than a 2-week culture, those viruses both labeled the sciatic nerve cells (Figure 3B). Out of eight infected sciatic nerves, we found neuronal cell bodies in three sciatic nerves (Figure 3C). Those cells were all identified as neuronal cells by staining for *Peripherin*, a marker of peripheral neurons (Escurat et al., 1990; Figure 3D). All this evidence indicated that there were neuronal cells in the cultured sciatic nerves.

### Neuronal Stem-Like Cells Identified by Single-Cell Sequencing Analysis

**FIGURE 4:**
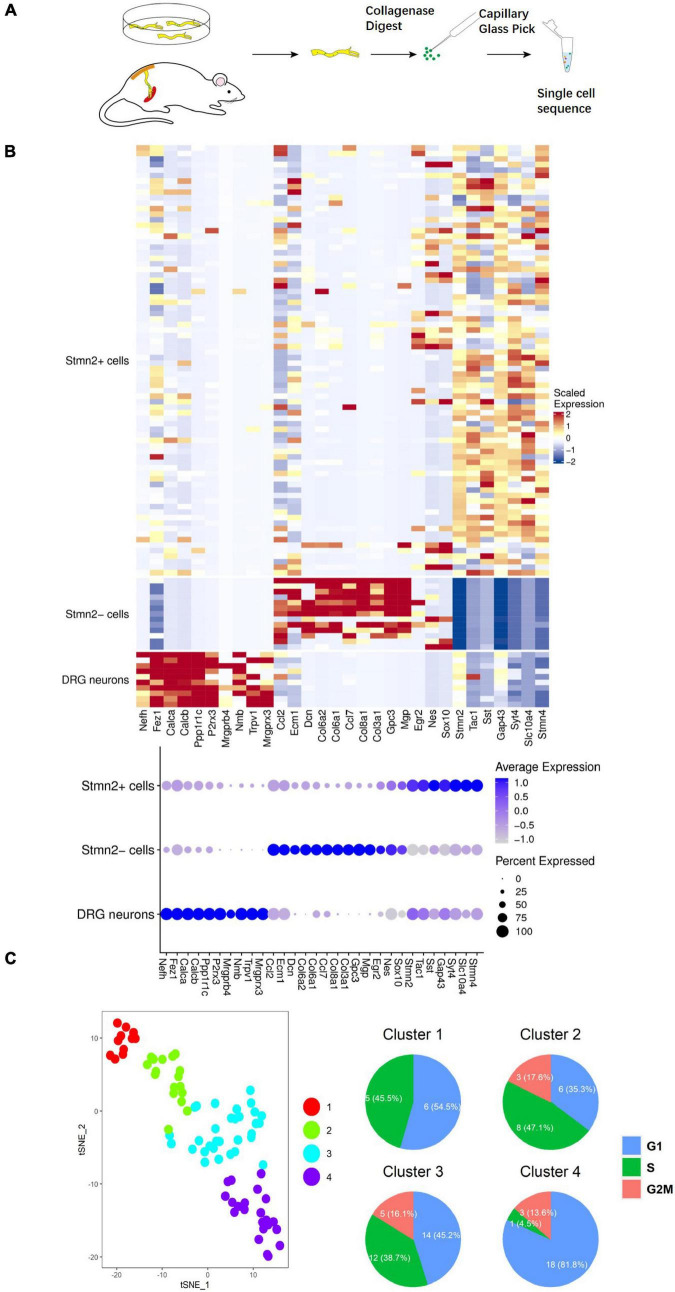
Single-cell sequencing of the neuronal cells marked by AAV2/9 virus in sciatic nerves of adult rats. **(A)** Single cell acquisition model of virus-labeled neuronal cells. **(B)** Single-cell sequencing of virus-marked cells. Single-cell sequencing of neuronal cells was conducted after 2 weeks of *hSYN*-promoter AAV2/9 virus infection. The hot map represents the transcriptome similarities between 78 *GFP*^+^ *Stmn2*^+^ cells, 13 *GFP*^+^ *Stmn*2^–^ cells, and 10 DRG cells. *GFP*^+^ cells were dissected from nerves injected with *hSYN*-promoter GFP AAV2/9 virus. DRG neurons were dissected from adult rat DRGs *in vivo*. A total of 84 cells from cultured sciatic nerves, seven cells from virus-injected sciatic nerves *in vivo*, and 10 cells from DRG neurons. **(C)** t-SNE map represents the subcluster analysis of the 78 *GFP*^+^ cells that expressed high levels of *Stmn2*. The four colors represent four different clusters. The pie diagrams represented the cell cycle analysis of those cells. A total of 74 cells from cultured sciatic nerves and four cells from virus-injected sciatic nerves *in vivo*. See also Supplementary Figure 1.

To prospectively identify the labeled cells enables us to directly examine their properties at the molecular level. Next, we conducted single-cell sequencing after the 2-week culture (Figure 4A). A total of 91 cells were dissected from sciatic nerves with GFP-positive cells *in vitro* and *in vivo* (seven cells were from *in vivo*) (Supplementary Figure 1). Ten DRG neurons were used as a positive control. Unsupervised clustering analysis assessed the separation of DRG neurons, *Stmn2*^+^ cells, and *Stmn2*^–^ cells (Blondel et al., 2008; Figure 4B). The *Stmn2*^+^ cells were different from the DRG neurons and expressed many neural stem cell markers such as *Nestin* and *Sox 10*. *Stmn2*-negative cells expressed many fibroblast cell markers such as *Col6a2* and *Col8a1*.

**FIGURE 5:**
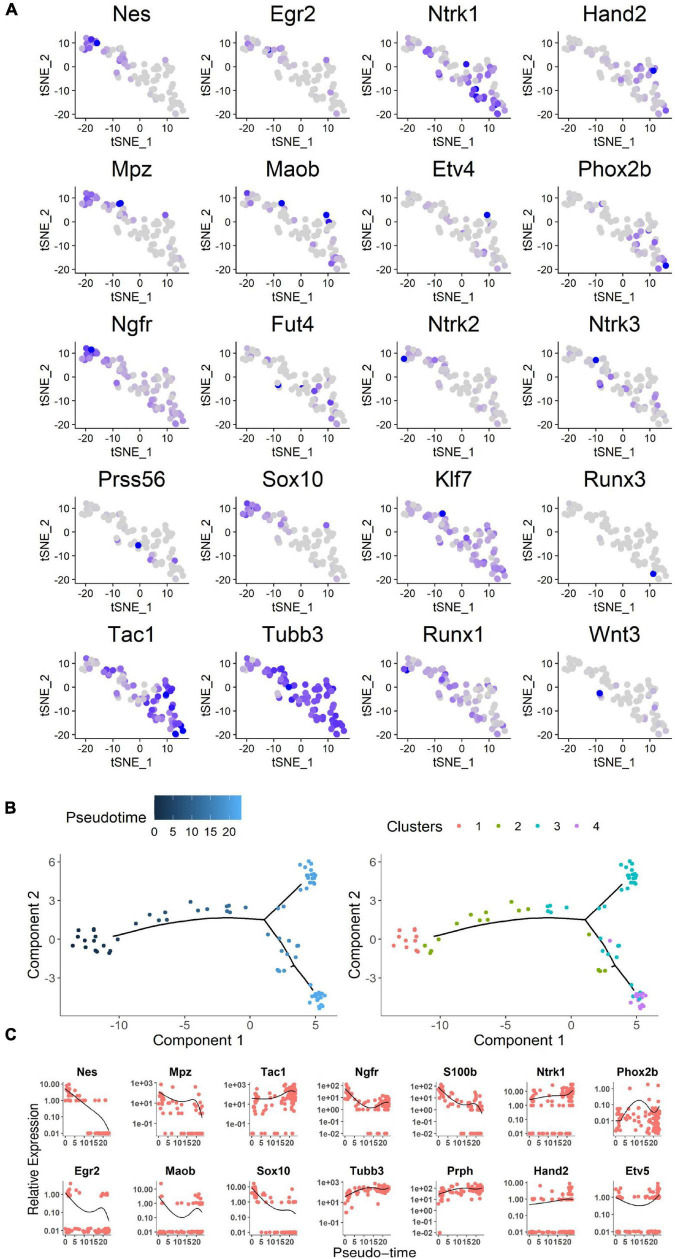
Analysis of neuronal cell single-cell sequencing data. **(A)** Expression profile of neural crest differentiation-related genes in the t-SNE map. Blue gradient represented the level of gene expression. **(B)** Expression profile of representative genes along the pseudo-time. Cells were ordered on the *x* pseudo-time axis by development of neural stem cells. Relevant gene expression was shown on the *y* axis as transcript counts (the black line was the running mean of expression with a window size of all cells). Each dot represents a cell. As show on the right, cells are highlighted in the same subcluster color gradient as in Figure 4C. **(C)** The pseudo-time genes expression level based on expression gradient. The chosen genes were representative examples of pseudo-timed differential expression along the transition from proliferation to differentiation for neural crest stem cells, progenitors, and neurons. See also Supplementary Figure 1.

*Stmn2* is highly expressed during neuronal stem cell development (Sugiura and Mori, 1995). Overall, 78 *Stmn2*^+^ cells were separated into four clusters by further unsupervised clustering analysis (McDavid et al., 2013). Cell division was more active in clusters 2 and 3 than in clusters 1 and 4, as indicated by the cell cycle analysis (Nestorowa et al., 2016; Figure 4C). It indicated that some of those cells were proliferating. Cells in clusters 1 and 2 showed high level expression of *Egr2* (*Krox20*), *Nestin*, and *Mpz* (*Protein 0*), which are markers of neural crest cells during the early fetal period (Dupin and Sommer, 2012). Cells in clusters 3 and 4 showed high expression of *Tac1* and *Tubb3*, which encode markers of mature neurons (Jiang and Oblinger, 1992; Hokfelt et al., 2001; Figure 5A). Several genes involved in differentiation of neuronal stem cells were identified in the clusters. *Ntrk1* (Molliver and Snider, 1997) mainly marks small-diameter and nociceptive neurons which are different from progenitor cells with highly expression of *Klf7* (Lei et al., 2005) and Runx1 (Marmigere et al., 2006); *Ntrk2* (Farinas et al., 1998) mainly marks large to medium-diameter and mechanoreceptive neurons which are different from progenitor cells with highly expression of *Wnt3* (Krylova et al., 2002) and *Runx3* (Inoue et al., 2002); *Ntrk3* (Moqrich et al., 2004) mainly marks large-diameter proprioceptive neurons. Most of those cells had high level expression of *Ntrk1*. It indicated those neuronal cells are involved in the sensory pathway of pain.

Although it was difficult to clearly separate discrete subpopulations by temporal gene expression patterns, developmental progression could be characterized by differential expression of specific neural crest stem cell genes (Bronner and Simoes-Costa, 2016). Moreover, a method called Monocle was used to order single cells through labeled cell differentiation and to construct a tree-like structure of the whole lineage differentiation trajectory (Figures 5B,C). Expression of genes that are hallmarks of the early state of neural crest stem cells (such as Egr2, Mpz, Nestin) was high in the earliest developmental stages and had disappeared in mature neurons. Sox10, which is involved in the differentiation of the neural crest stem cells (Kelsh, 2006), was also expressed in the early stages. From the early stages to mature neurons, genes expression associated with neurons was upregulated (for instance, Tubb3 and Tac1; Figure 5C). Among those 78 GFP^+^ *Stmn2*^+^ cells, four cells were from *in vivo* while the others from *in vitro* cultures. Those *in vivo* cells presented a similar condition to the *in vitro* cells as the lineage differentiation trajectory or the cell division analysis indicated (Supplementary Figure 1).

Together, these findings suggested that the neuronal cells marked by AAVs in adult sciatic nerves contain neurons and neuronal stem-like cells which could proliferate.

### Neuronal Stem-Like Cells Marked by *Nestin* Antibody in Adult Sciatic Nerve

**FIGURE 6:**
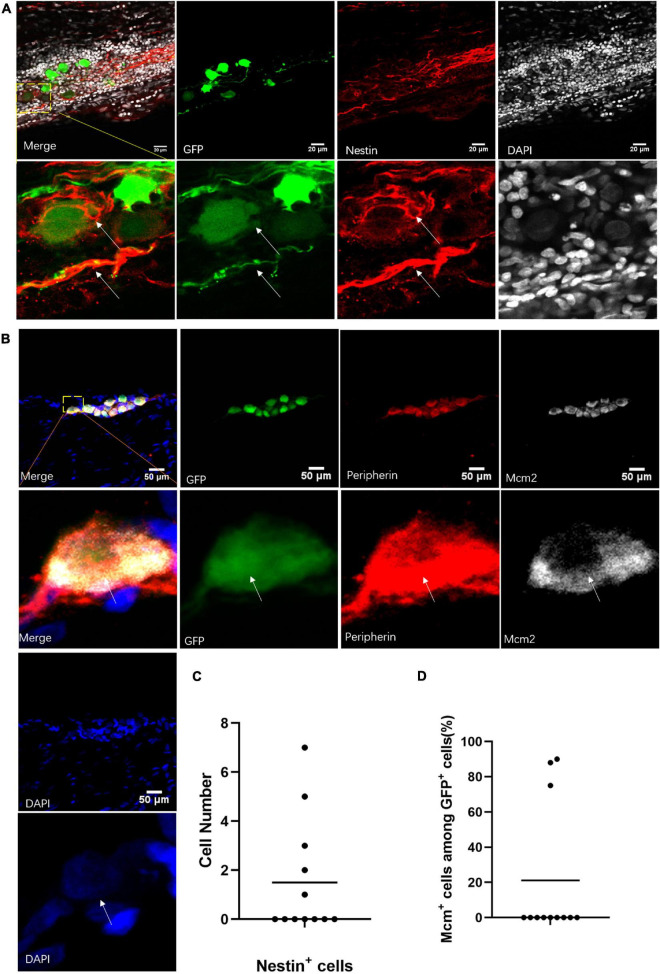
Neuronal stem-like cells in sciatic nerves of adult rats. **(A)** Stem cell marker staining of neuronal cells in sciatic nerves after virus injection. Immunofluorescent staining for *Nestin* (red) and *DAPI* (gray) of *GFP*^+^ (green) neuronal cells after infection of *hSYN*-promoter *GFP* AAV2/9 virus. *n* = 12 (*GFP*^+^ sciatic nerves), *n* = 5 (*Nestin*^+^ sciatic nerves). Scale bar, 20 μm. **(B)** Neuronal cells in a phase of division. Confocal images of immunostaining of *Mcm2* (gray), *Peripherin* (red), and *DAPI* (blue) in *GFP*^+^ (green) neuronal cells. The sciatic nerves were infected by *hSYN*-promoter *GFP* AAV2/9 virus. *n* = 12 (sciatic nerves with *GFP*^+^ neuronal cells), *n* = 3 (*Mcm2*^+^ sciatic nerves). Scale bar, 50 μm. **(C)** The quantification of the number of *Nestin*^+^ cells in the sciatic nerves with neuronal cells. The sciatic nerves with neuronal cells were infected by *hSYN*-promoter *GFP* AAV2/9 virus (*n* = 5 of 12 which contain *Nestin*^+^ cells, the other without *Nestin*^+^ cells). **(D)** The quantification of the percentage of *Mcm2*^+^ cells among all *GFP*^+^ neuronal cells in the sciatic nerves. The cultured sciatic nerves were infected by *hSYN*-promoter *GFP* AAV2/9 virus. The circle points represent the percentage of *Mcm2*^+^ cells among *GFP*^+^ cells (*n* = 3). The white arrow points to the positive cells.

To verify the single-cell sequencing data, we used immunobiological markers to identify the cell types of the neuronal cells labeled by virus in the sciatic nerves. Some of those cells could be marked by *Nestin* antibody in the sciatic nerves after 2 weeks of virus injection (Figure 6A). Out of 12 infected sciatic nerves with neuronal cells, we found *Nestin*^+^ cells in five sciatic nerves (Figure 6C). Importantly, the *GFP*^+^ cells were also positive for cell proliferation marker *Mcm2* (Maslov et al., 2004; Figure 6B). Those *Mcm2*^+^ *GFP*^+^ *Peripherin*^+^ cells were found in 3 of 12 infected sciatic nerves with neuronal cells (Figure 6D). This evidence suggested that neuronal stem-like cells are proliferating in adult sciatic nerves.

### Proliferation of Neuronal Stem-Like Cells in the Sciatic Nerves of Adult Rats

**FIGURE 7:**
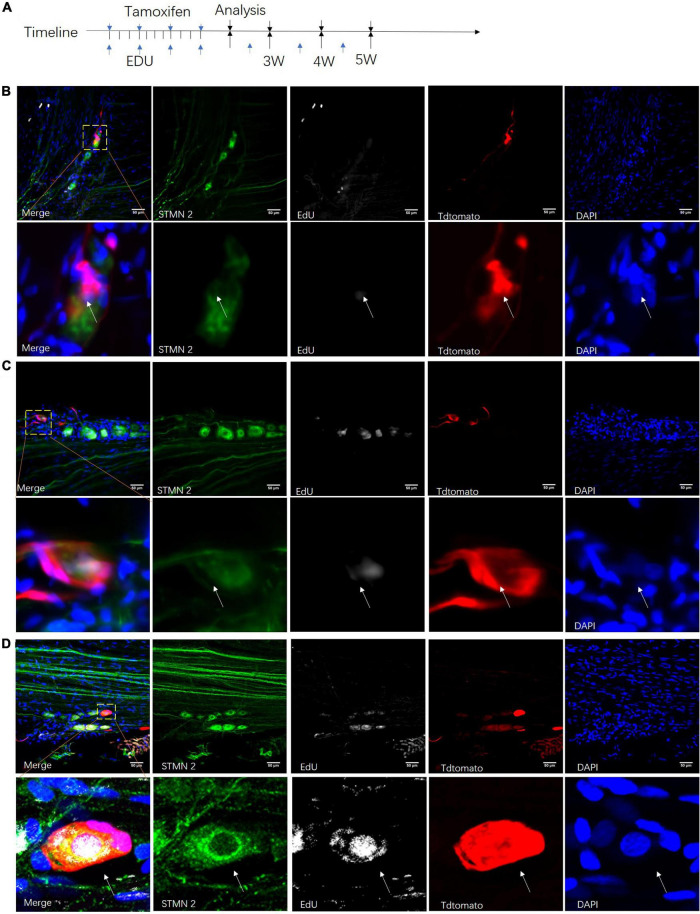
Proliferation of neuronal stem-like cells in the sciatic nerves of transgenic rats. **(A)** Adult *Nestin-CreERT*^2^*::Tdtomato* rats were given injections of tamoxifen and EdU at different time points for clonal lineage-tracing analysis. **(B–D)** Confocal images of Stmn2-labeled cells (green) co-labeled with *TdTomato* (red), EdU (gray), and *DAPI* (blue) after tamoxifen and EdU injection for 3–5 weeks. Scale bar, 50 μm. *n* = 5 for each time point. The white arrow points to the positive cells.

To understand the fate of those neuronal stem-like cells in the sciatic nerves, we constructed a *Nestin-CreERT*^2^ rat line which used the same strategy as a *Nestin-Cre* mouse model (Dubois et al., 2006) for sequential observation. Transgenic rats carrying a *TdTomato* transgene whose expression was dependent on *Cre*-mediated recombination were used to create offspring and report the expression of *Nestin*. At different time points after tamoxifen and EdU intraperitoneal injection, we used *Stmn2* antibody to identify and quantify cells labeled with *TdTomato* (Figure 7A). We observed obvious neuronal stem cells and neurogenesis in the dentate gyrus of those rats, consistent with prior studies (Kuhn et al., 1996; Kempermann et al., 2015; Supplementary Figure 2). At the same time we injected saline solution in the control rats; no detectable *TdTomato*^+^ cells were found in those rats. We detected a number of *TdTomato*^+^ cells which were co-labeled with *Stmn2* and EdU in the sciatic nerves (Figures 7B–D). The *TdTomato*^+^ *Stmn*2^–^ cells, *TdTomato*^–^ *Stmn2*^+^ cells, and *TdTomato*^+^ *Stmn2*^+^ cells could be detected in one cell cluster in the sciatic nerves after 5 weeks (Figure 7D).

**FIGURE 8:**
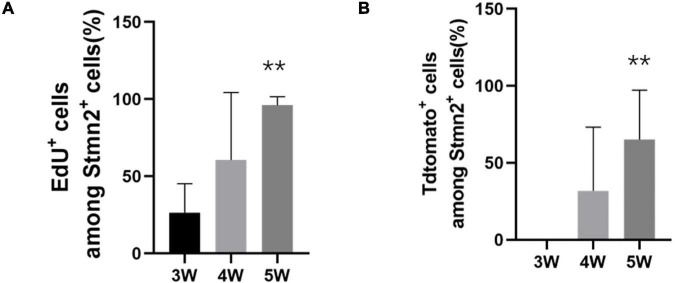
The quantification of proliferation of the neuronal stem-like cells. **(A)** The quantification of the percentage of EdU^+^ cells among all Stmn2^+^ cells in the sciatic nerve. Values represent mean ± SEM (*n* = 5 sciatic nerves for each time point; **p < 0.05; one-way ANOVA with Dunnett’s multiple comparisons test). **(B)** Quantification of the proportion of TdTomato^+^ cells among all Stmn2^+^ cells in the sciatic nerve. Values represent mean ± SEM (*n* = 5 sciatic nerves for each time point; **p < 0.05; one-way ANOVA with Dunnett’s multiple comparisons test).

In the first 3 weeks after tamoxifen injection, most of those cells were set aside in quiescence without being co-labeled with EdU (Figure 8A). And *TdTomato*^+^ cells were not co-labeled with *Stmn2* (Figure 8B). At 4 or more weeks, more and more *TdTomato*^+^ cells were co-labeled with *Stmn2* and EdU. And *Stmn2*^+^ cells without EdU labeling had disappeared. In the first 3 weeks, we were hardly able to detect the *Stmn2*^+^ cells co-labeled with *TdTomato*. Although very rare, we could notice *TdTomato*^+^ *Stmn2*^–^ cells co-labeled with EdU. This evidence suggested that neuronal cells in different proliferation stages are together in sciatic nerves.

Taken together, this evidence indicated that the neuronal stem-like cells could proceed proliferation in the sciatic nerves of adult rats.

## Discussion

In the current study, we explored the cell types in the sciatic nerve of adult rats. The results showed that neuronal cell bodies distribute along the sciatic nerves from distal to proximal. In addition, both in the crush model rats or in normal rats, neuronal cells existed (Figure 1). At first, we hyalinized the sciatic nerve by a sorbitol-based optical clearing method which called ScaleS (Hama et al., 2015). It helped us to obtain stable sciatic nerve preservation for immunochemical labeling. And then, optical imaging of the normal and crushed sciatic nerve tissue for *Stmn2* (Shin et al., 2014) was used to observe neuronal cells inside the sciatic nerves. It clearly presented the neuronal cell bodies in both crushed and normal sciatic nerves.

*Stmn2* is enriched in the growth cones of the developing neurons as a microtubule destabilizing factor (Stein et al., 1988). It is a neuron-specific protein with a genetic control element, *NRSE/RE-1*. *NRSE/RE-1* prevents the expression of *Stmn2* by binding the repressor/silencer protein *NRSF/REST*, which presents in many non-neuronal cell lines and tissues (Mori et al., 1992; Saffen et al., 1999). In the peripheral nervous system, Stmn2 is expressed by neurons but not by glia. Otherwise, the diameter of most of those cells is about 25–45 μm. So the mean size of those cells is the same as the rat DRG neurons which is about 29 μm (Swett et al., 1991).

*NeuN* has been considered to be a reliable biomarker of mature neurons for more than two decades (Mullen et al., 1992). In nearly all parts of the vertebrate nervous system, *NeuN* is distributed in the nuclei of mature neurons and highly conserved among species (Wolf et al., 1996; Duan et al., 2016). A small number of those neuronal cells could be stained by *NeuN* in the sciatic nerves of adult rats indicating that some of those cells are mature neurons. Peripherin is established as a type III IF protein which could bind together with either NF proteins or vimentin assemble filaments (Escurat et al., 1990). Many studies using biochemical methods have shown that the expression of peripherin is localized in the neurons of the PNS (Yuan et al., 2012). We used single-cell sequencing to identify those neuronal cells marked by AAV2/9 virus. Some of those cells expressed the markers of neurons such as *Tac1* and *Tubb3*, which are markers of mature neurons (Jiang and Oblinger, 1992; Hokfelt et al., 2001) and some expressed the markers of neuronal stem cells such as *Egr2* (*Krox20*), *Nestin*, and *Mpz* (*protein 0*), which are markers of migrating neural crest cells during the early fetal period (Dupin and Sommer, 2012). Furthermore, we found most of those cells expressed *Ntrk1*, *Klf7*, and *Runx1* via single-cell sequencing analysis. *Ntrk1* is required for the development and survival of nociceptive sensory and sympathetic neurons, accounting for 80% of the total DRG population (Molliver and Snider, 1997). *Klf7* specifically regulates *Ntrk1* gene expression and is required for the development of a subset of nociceptive sensory neurons (Lei et al., 2005). *Runx1* promotes axonal growth and is selectively expressed in *Ntrk1*^+^ sensory neurons (Marmigere et al., 2006). All this evidence demonstrated that neuron-like cells, some of which were mature neurons, were found in rat sciatic nerves. And most of them would differentiate into nociceptive sensory neurons.

It was difficult to identify these cells in every sciatic nerve as they were only present in part of the sciatic nerves. Five of sixteen crushed sciatic nerves and five of eighteen normal sciatic nerves contained neuronal cells. In the cultured sciatic nerves, 6 of 12 sciatic nerves contained those cells. The current study has not defined the source of those neuronal cells. We could not find the neuronal cells at the same points in all the sciatic nerves of different rats. Those cells may be sensory neurons and stem cells which move to the sciatic nerve during the embryonic development. There may be a leak of some neurons and neuronal stem cells in the sciatic nerve from the DRG. In a future study, tracing those cells in the sciatic nerve since embryonic development is needed.

It should be noted, however, that those cells may also transdifferentiate from other cells such as glia cells. The concept of neural crest stem cells (NCSCs) was introduced by a milestone study published by Morrison et al. (1999). *In vivo*, fetal sciatic nerve NCSCs were shown to give rise to glia and nerve fibroblasts in the genetic fate mapping of mice (Joseph et al., 2004). They were able to give rise to peripheral neurons and glia when they were transplanted into the hindlimb bud somite of chick embryos. Schwann cell precursors (S) are direct derivatives of the truncal and cranial neural crest cells (Jessen and Mirsky, 2005). SCPs keep certain molecular characteristics of neural crest cells and remain multipotent to produce a variety of cell types (Morrison et al., 1999). So the SCPs may be a kind of long-lasting nerve-associated pool of neural crest-like cells that reside in the sciatic nerve. NCSC-like cells in postmigratory targets of the embryonic neural crest cells are discovered in the sciatic nerve, DRG, gut, and skin (Shakhova and Sommer, 2010). Although the postmigratory NCSCs from different regions can generate neuronal, glial, and non-neural cell types, their multipotency is limited by the environment (Kruger et al., 2002). Some subpopulations of NCSCs with high plasticity and a sphere-forming capacity persist in the sciatic nerve during the late fetal stage to adulthood *in vitro*, it is technically challenging to demonstrate that those post-migratory neural crest cells could maintain their multipotent properties and developmental plasticity *in vivo* after birth (Bronner and Simoes-Costa, 2016; Parfejevs et al., 2018). So we hope that some new methods will be introduced to clarify the fate of neurons and neuronal stem-like cells in the sciatic nerves of adult rats in the future.

*Stmn2* and *NeuN* staining and the morphological feature of those cells suggested part of those cells are neurons. The analysis of the single-cell sequencing data of those cells indicated there are neuronal stem-like cells other than neurons in the sciatic nerves. According to the expression of differentiation markers, we could identify neuronal stem-like cells at different development stages including neuronal stem cells, progenitors, and mature neurons in the sciatic nerves as the pseudo-time analysis indicated. Moreover, most of those new-born neurons were nociceptive neurons as the single-cell sequencing indicated.

In *Nestin-CreERT*^2^ rats, our study indicated the proliferation of neuronal stem-like cells in the sciatic nerves. We only detected *TdTomato*^–^ *Stmn2*^+^ cells in the sciatic nerve in the first 3 weeks. But we rarely detected *TdTomato*^+^ *Stmn2*^–^ EdU^+^ cells. It showed the quiescent condition of the neuronal stem-like cells at first. Most *TdTomato*^+^ *Stmn2*^+^ cells were positive for EdU staining after 4–5 weeks. It suggests that most of those cells are from the proliferation of neuronal stem-like cells.

In postnatal parasympathetic ganglia of the head or gut, differentiation of SCPs is a programmed organizational process that occurs at a defined time and place. In adults, neural crest-derived cells show injury and stress responses, likely involving dedifferentiation and *in vivo* reprogramming to acquire a new cell fate (Parfejevs et al., 2018). In the central nervous system, cell fate and differentiation of hippocampal and V-SVZ NSCs into mature functional neurons proceed through a clearly defined set of stages. These stages progress from type 1 cells, to type 2 cells, to type 3 cells in the hippocampal or from type B1 cells, to type C cells, to type A cells in the V-SVZ, which undergo final maturation into functional neurons (Kempermann et al., 2015; Lim and Alvarez-Buylla, 2016). Unlike the SVZ and SGL, only in the context of the homeostatic adaptive response to chronic hypoxia, is the carotid body (CB) germinal center turned on. Upon exposure to hypoxia, activation of neuronal stem cell proliferation is paralleled by a rapid conversion of the new born cells into intermediate progenitors, which give rise to mature *TH*^+^ glomus cells (Pardal et al., 2007). According to our study of neuronal stem-like cells in the sciatic nerves, many reasons induced the differentiation of those cells other than injury and stress. On the basis of the wide location of those cells in adult sciatic nerves in various time points, the proliferation of neuronal stem-like cells was widespread along the sciatic nerves. We will explore the reason of the existence and proliferation of those neurons and neuronal stem-like cells in a future study.

Taking the above into consideration, our results indicate that there are neuronal cells including neurons and neuronal stem-like cells which can proliferate *in vivo* in the sciatic nerves of adult rats.

## Materials and Methods

### Resource Availability

#### Lead Contact

Further information and requests for resources and reagents should be directed to and will be fulfilled by the lead contact, XG (nervegu@ntu.edu.cn).

#### Materials Availability

All unique/stable reagents generated in this study are available from the lead contact without restriction.

#### Data and Code Availability

The 101 single-cell sequencing data (sample gene expression in FPKM) are available on jianguoyun^1^ for the bioinformatic analysis. The raw sequence data reported in this article have been deposited in the Genome Sequence Archive (Genomics, Proteomics and Bioinformatics, 2021), National Genomics Data Center (Nucleic Acids Research, 2021), and China National Center for Bioinformation/Beijing Institute of Genomics, Chinese Academy of Sciences (GSA: CRA005995) that are publicly accessible at https://ngdc.cncb.ac.cn/gsa. The temporary shared URL can be found here: https://ngdc.cncb.ac.cn/gsa/s/2i4aNSsi.

### Experimental Model and Subject Details

#### Animals

Sprague-Dawley (SD) rats (8 weeks old) from the laboratory animal center of Nantong University were used. A transgenic *Nestin-CreERT*^2^ rat line was purchased from Beijing Biocytogen Co., Ltd. Rats were 8 weeks old when they were used in the study. Experimental and control rats of both genders were littermates housed together before the experiment. All the experiments used the left side nerve except those in Figures 4, 7 in which both sides were used. A total of approximately 151 rats were used. All animal procedures were performed in accordance with Institutional Animal Care guideline of Nantong University, and were ethically approved by the Administration Committee of Experimental Animals, Jiangsu Province, China.

### Method Details

#### ScaleS and Immunohistology

The transparency procedure was the same as described in other studies that used ScaleS. Briefly, the epineural sheath must first be peeled away from the sciatic nerve sample. The permeability of a sample was enhanced by incubation for 12 h in ScaleS0 solution (20% sorbitol, 5% glycerol, 1 mM methyl-β-cyclodextrin, 1 mM γ-cyclodextrin, 1% N-acetyl-L-hydroxyproline, and 3% DMSO). Second, the permeable (adapted) sample was incubated sequentially in ScaleA2 (10% glycerol, 4 M urea, 0.1% Triton X-100 for 36 h), ScaleB4 (0) (8 M urea for 24 h), and ScaleA2 (for 12 h) for permeabilization/clearing. These urea-containing and salt-free ScaleS solutions gradually clear the sample. Then, after descaling with PBS(–) wash for at least 6 h, the sample was incubated for 36 h with a fluorescence-labeled primary antibody (Ab) (direct IHC) or a primary Ab and then a fluorescence-labeled secondary Ab (indirect IHC) in an AbScale solution [PBS(–) solution containing 0.33 M urea and 0.1–0.5% Triton X-100]. Before refixation with 4% PFA, we applied an AbScale rinse solution to the sample twice, for 2 h each time [0.1 × PBS(–) solution containing 2.5% BSA, 0.05% (w/v) Tween-20]. Finally, the immune-stained sample was optically cleared by incubation in ScaleS4 for more than 16 h (40% sorbitol, 10% glycerol, 4 M urea, and 0.2% Triton X-100).

#### EdU and Tamoxifen Injection and Labeling

A stock solution of 10 mg/ml of EdU (Invitrogen, A10044) was prepared in normal saline solution (0.9%). A stock solution of 55 mg/ml of tamoxifen (Sigma, T5648) was prepared in a 5:1 solution of corn oil: ethanol at 37°C in a water bath kettle with occasional vortexing overnight. EdU (10 mg/kg) was injected in Nestin-CreER*T*^2^ rats (6–8 weeks old, 200 g) with tamoxifen injection (55 mg/kg) at the time points shown in Figure 7A. After secondary antibody staining, EdU staining was performed according to the manufacturer’s guidelines (Click-iT EdU Alexa Fluor 647 Imaging Kit, Invitrogen).

#### Rat Sciatic Nerve Culture Preparation and Treatment

We cultured sciatic nerves from adult 8-week-old SD rats in 10 cm plates (Corning) coated with poly-D-lysine (Sigma) and laminin (Sigma). The sciatic nerve was cut from below the DRG (omitting all DRG tissue) to the nerve ending. We carefully peeled away the epineural sheath in cold PBS. The collected sciatic nerves were plated as a line at a density of approximately eight sciatic nerves per dish and kept for 20 min at 37°C (making sure they were fixed on the plates), and a Neurobasal medium (Invitrogen) supplemented with 2% (vol/vol) B27 (Invitrogen) and 25 ng/mL of nerve growth factor (Sigma) was added. Cultured sciatic nerves were maintained for 1 day prior to injection. The culture medium was discarded before the injection. The virus (20 μl/8 nerves, with virus titers of approximately 2.50E + 13) was dropped slowly and uniformly onto the sciatic nerve and then incubated for 2 h before adding the culture medium (carefully ensuring that the sciatic nerve did not dry out). Then, the sciatic nerve was cultured for 2 weeks before observation to allow for GFP expression from the sciatic nerve, where the cell bodies were placed. For more than 1-month cultures, the medium was changed every 5 days. We examined neurons using a fluorescence microscope.

#### Animal Surgery

For the sciatic nerve lesion experiment, the adult rats (8 weeks old) were anesthetized by intraperitoneal injection with 85 mg of trichloroacetaldehyde monohydrate, 42 mg of magnesium sulfate, and 17 mg of sodium pentobarbital. We exposed the sciatic nerve at the sciatic notch by making a small incision. The sciatic nerve at 10 mm above the bifurcation into the tibial and common fibular nerves was crushed with a forceps at a force of 54 N three times (a period of 10 s for each time). The crush site was marked with a size 10-0 nylon epineural suture. The muscle and skin were then sutured. For the control rats (8 weeks old), the sciatic nerve was exposed but left uninjured. After surgery, the wound was closed, and the rats were allowed to recover for 2 h.

For the sciatic nerve AAV injection, we anesthetized the adult rats (8 weeks old) with an intraperitoneal injection of complex narcotics (85 mg trichloroacetaldehyde monohydrate, 42 mg magnesium sulfate, and 17 mg sodium pentobarbital) and carefully opened the skin and muscle to expose the sciatic nerve. Then 10 cm capillary glass tubes (Sutter Instrument, Novato, CA, United States) were pulled using a micropipette puller (model 720, David KOPF Instruments, Tujunga, CA, United States). The tips of the pulled tubes were pinched with forceps to create pipettes with an external diameter of approximately 10 μm. A 2.5 μl volume of AAV2/9 (virus titers were approximately 2.50E + 13) was gradually injected into the sciatic nerve with one pump of a micro-syringe pump at a rate of 1 μl/min (Stoelting Instruments). The needle tip was inserted into the epineural sheath, and the drops caused it to plump up. After three injections at three different sites along the sciatic nerve, the wound was closed, and the rats were allowed to recover for 2 h.

For the tail AAV injection, we placed the 8-week-old rats in a restraint device. The tail was stabilized between the investigator’s thumb and forefinger. To soften the skin, the tail was prepared in 40°C water for 5 min and then sterilized by 70% ethanol. The injection started at the distal part of the tail with an insulin syringe. With the tail under tension, the needle was inserted approximately parallel to the vein at a depth of at least 3 mm. A 30 μl solution of AAV-PHP.S (the virus titers were approximately 2.50E + 13) mixed into 150 μl of PBS was slowly injected over 3 min. After the vein blanched, the needle was kept in position for 1 min. The rats were allowed to recover for 2 h and then returned to their home cages.

#### Preparation of Individual Cells From Adult Rat Sciatic Nerve

We conducted single-cell sequencing experiments on SD rats subjected to *hSYN-GFP* AAV2/9 and AAV2/9-*hEF1a-GFP* virus sciatic nerve injection *in vivo* and *in vitro*. The rats were euthanized by cervical dislocation, and the sciatic nerve was immediately immersed in ice-cold Dulbecco’s Phosphate-Buffered Saline (DPBS, Corning). The dissected sciatic nerve was cut from the distal end of the DRG to the end of the sciatic nerve (leaving out the DRG) and we carefully peeled away the epineural sheath in cold PBS. Collected sciatic nerves were cut into pieces under a fluorescence microscope. The *GFP*^+^ piece was collected and incubated in Hibernate A (BrainBits) containing papain (100 U; Sigma) at 37°C for 2 h with intermittent flicking. After removing enzymes, the collected pieces were trypsinized for 20 min at 37°C. The tissue was triturated into an individual cell suspension using a 1 ml pipette. We removed the trypsase and cellular debris with three rounds of mild centrifugation at 1000 × *g* and a Hibernate A minus Ca^2+^ and Mg^2+^ wash (BrainBits). The individual cell suspension was plated into a glass-bottom plate and collected using glass pipettes under a fluorescence microscope. The glass tip was broken off and left in each PCR tube containing lysis buffer (Vazyme Biotech) with water (2.4 μl), RNase-free DNase (0.2 μl), and murine origin RNase inhibitor (0.25 μl).

#### Cell Preparation

Sprague-Dawley rats with *hSYN* or *CMV*-promoted GFP AAV2/9 injection in the sciatic nerve were used for electrophysiology experiments. Rats were euthanized by cervical dislocation, the sciatic nerves were immediately immersed into ice cold Dulbecco’s Phosphate-Buffer Saline (DPBS, Corning). The dissected sciatic nerve was cut from the DRG to the end of the sciatic nerve containing the trifurcation among tibial sural and peroneal branches. And then we carefully peeled away the epineural sheath in cold PBS. Collected sciatic nerves were cut into pieces under a fluorescent microscope. The GFP-positive piece was picked up and incubated in Hibernate A (BrainBits) containing papain (100 U; Sigma) at 37°C for 2 h with intermittent flicking. After the removal of enzymes, the collected pieces were trypsinized for 20 min at 37°C. The tissue was triturated into individual cell suspensions by a 1 ml pipette. Enzymes and cellular debris were removed with multiple rounds (three times) of mild centrifugation at 1000 *g* and washing with Hibernate A minus Ca^2+^ and Mg^2+^ (BrainBits). The individual cell suspension was plated onto a glass bottom plate.

#### Library Preparation, Clustering, and Sequencing

We used the Vazyme method, followed by cDNA amplification as described below. Whole transcriptome amplification was performed using the Discover-scTM WTA Kit V2 (Vazyme, N711). First, 101 active cells were isolated and transferred into a lysis buffer. Then, mRNA was copied into first-strand cDNA using Discover-sc Reverse Transcriptase and oligo(dT) primer. At the same time, we added a special adapter sequence to the 3′ end of the first-strand cDNA. Full-length cDNA enrichment was performed by PCR, and the products were purified by VAHTSTM DNA Clean Beads (Vazyme, N411). Next, we performed quality control using the WTA cDNA. The cDNA concentration was measured using a Qubit DNA Assay Kit in a Qubit 3.0 Fluorometer (Life Technologies, Carlsbad, CA, United States). DNA fragment size was tested using an Agilent Bioanalyzer 2100 system (Agilent Technologies, Palo Alto, CA, United States). A total of 1 ng of qualified WTA cDNA product per sample was used as input material for the library preparation.

We generated sequencing libraries using the TruePrep DNA Library Prep Kit V2 for Illumina (Vazyme, TD503), following the manufacturer’s recommendations. First, cDNA was randomly fragmented by the Tn5 transposome at 55°C for 10 min at the same time as a sequencing adapter was added to the 3′ adenosine on the fragment. After tagmentation, the stop buffer was added directly into the reaction to end tagmentation. PCR was performed, and the products were purified with VAHTSTM DNA Clean Beads (Vazyme, N411). We conducted preliminary quantification of the library concentration using a Qubit DNA Assay Kit in Qubit 3.0. Insert size was assessed using the Agilent Bioanalyzer 2100 system, and if the insert size was consistent with expectations, it was more accurately quantified using qPCR with the Step One Plus Real-Time PCR system (ABI, United States).

We identified the neuronal cells via *Stmn2* expression. We identified 74 *Stmn2*^+^ cells *in vitro* and 4 *Stmn2*^+^ cells *in vivo* using qPCR before sequencing. Thirteen *Stmn2*^–^ cells were identified as negative control. Ten DRG neurons were identified as positive control.

Clustering of the index-coded samples was performed on a cBot Cluster Generation System (Illumina) according to the manufacturer’s instructions. After cluster generation, the library preparations were sequenced on an Illumina HiSeq X Ten platform with a 150 bp paired-end module. The single-cell sequencing was executed by Vazyme Co., Ltd.

#### Bioinformatic Analysis

Samples were then normalized by down-sampling to a minimum number of 101 transcripts per cell for the clustering analyses or a minimum of 78 transcripts per cell for differential gene expression analyses. Cells with fewer transcripts were excluded from the analyses. The modularity optimization technique SLM was used for unsupervised cell clustering. We used t-SNE to place cells with similar local neighborhoods in high-dimensional space together. The analysis was executed by GENEDENOVO Co., Ltd.

### Quantification and Statistical Analysis

All statistical analyses were conducted using GraphPad Prism software (GraphPad Software, La Jolla, CA, United States). Data were presented as mean ± SEM (error bars). One-way ANOVA with Dunnett’s multiple comparisons test was used to evaluate if the difference between two different groups was statistically significant. ^**^p < 0.05 indicate statistically changes.

### Key Resources Table

See the table in the Supplementary Material.

## Data Availability Statement

The datasets presented in this study can be found in online repositories. The names of the repository/repositories and accession number(s) can be found below: Genome Sequence Archive (Genomics, Proteomics and Bioinformatics, 2021) in the National Genomics Data Center (Nucleic Acids Research, 2021), China National Center for Bioinformation/Beijing Institute of Genomics, Chinese Academy of Sciences with GSA: CRA005995 (https://ngdc.cncb.ac.cn/gsa).

## Ethics Statement

All animal procedures were performed in accordance with Institutional Animal Care guidelines of Nantong University, and were ethically approved by the Administration Committee of Experimental Animals, Jiangsu Province, China.

## Author Contributions

YL and XG conceived and designed the project. YL, SZ, and LZ conducted the experiments and analyzed all the data. YL, SZ, and XG wrote the manuscript. All authors contributed to the article and approved the submitted version.

## Conflict of Interest

The authors declare that the research was conducted in the absence of any commercial or financial relationships that could be construed as a potential conflict of interest.

## Publisher’s Note

All claims expressed in this article are solely those of the authors and do not necessarily represent those of their affiliated organizations, or those of the publisher, the editors and the reviewers. Any product that may be evaluated in this article, or claim that may be made by its manufacturer, is not guaranteed or endorsed by the publisher.

## Footnotes

1
https://www.jianguoyun.com/p/DfCTlGMQlOWDChjpoqsE
