# Supplementary material for: Identification of Neuronal Cells in Sciatic Nerves of Adult Rats
Source: Front Cell Neurosci. 2022 Mar 25;16:816814. doi: 10.3389/fncel.2022.816814 (PMC8991689; doi:10.3389/fncel.2022.816814)
Supplement: Supplementary file 1 [file Data_Sheet_1.docx]

**Supplementary figures and figure legends**


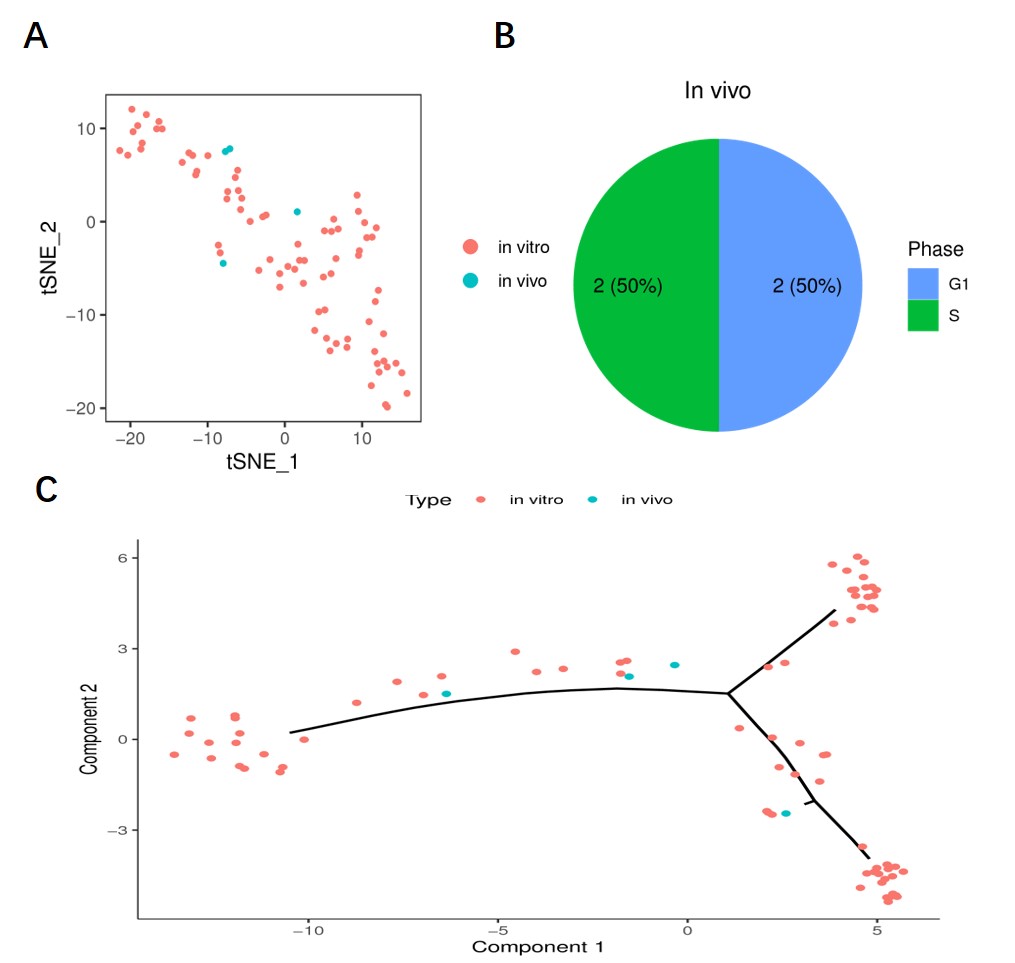


**Supplementary Figure 1, related to Figure 4C and Figure 5: Single-cell sequencing of the neural cells marked by AAV2/9 virus in sciatic nerves of adult rats in vivo.**

Single-cell sequencing analysis of four in vivo cells. The green dots in the clustering (A), cell cycle statistics (B), and the pseudo-time analysis (C) represent the cells dissected in vivo after virus injection for 2 weeks.


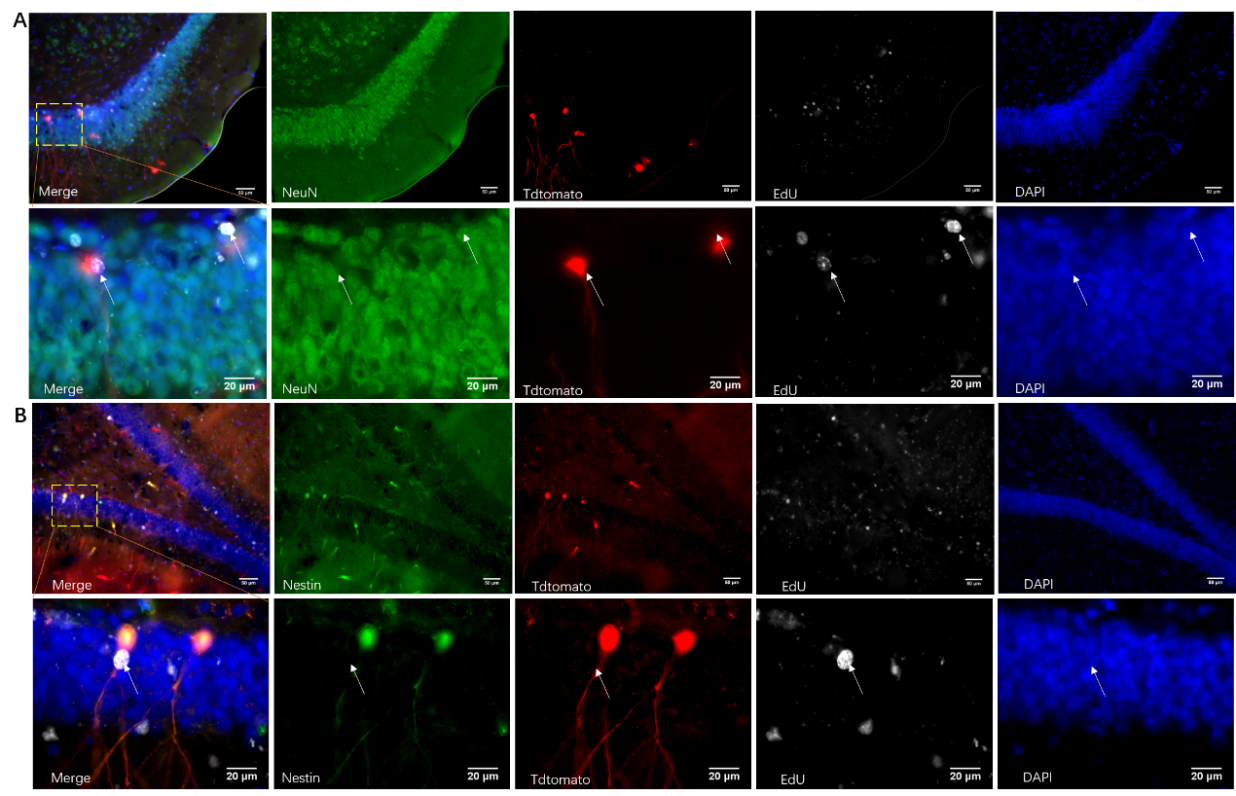


**Supplemental Figure 2, related to Figure 7: Neuronal stem cells and neurogenesis were readily detected by TdTomato with Nestin or NeuN staining, and EdU incorporation in the adult dentate gyrus of tamoxifen-induced *Nestin-CreER^T2^::TdTomato* rats.**

(A) As a positive control for neuronal stem cells and neurogenesis, we checked the hippocampus of *Nestin-CreER^T2^::TdTomato* rats induced by tamoxifen and EdU intraperitoneal injection in adult. We detected those cells in the rats after the drug injection as shown in Figure 7A. In each case the sections were stained with EdU (gray) and DAPI (blue) as well as antibodies against NeuN (green). We observed EdU^+^ NeuN^+^ neurons marked by TdTomato (white arrows) in the dentate gyrus. n=3. Scale bar, upper panels=50 μm, lower panels=20 μm.

(B) We also stained those sections with EdU (gray) and DAPI (blue) as well as antibodies against Nestin (green). We readily observed Nestin^+^ TdTomato^+^ neuronal stem cells (white arrows) in the dentate gyrus. n=3. Scale bar, upper panels=50 μm, lower panels=20 μm.

**KEY RESOURCES TABLE**

| REAGENT or RESOURCE | SOURCE | IDENTIFIER |
| --- | --- | --- |
| Antibodies | | |
| Stmn2 | ProteinTech | 10586-1-AP |
| NeuN | Millipore | MAB377 |
| GFP | Abcam | ab13970 |
| Peripherin | Aves | PER |
| Nestin | Aves | NES |
| Nestin | Chemicon | MAB353 |
| mCherry | Novus | NBP2-25158 |
| Goat anti-Mouse Secondary Antibody, Alexa Fluor 488 | Invitrogen/Thermofisher scientific | Cat # A32723 |
| Goat anti- Mouse Secondary Antibody, Alexa Fluor 555 | Invitrogen/Thermofisher scientific | Cat # A32727 |
| Goat anti- Mouse Secondary Antibody, Alexa Fluor 647 | Invitrogen/Thermofisher scientific | Cat # A32728 |
| Goat anti-Rabbit Secondary Antibody, Alexa Fluor 488 | Invitrogen/Thermofisher scientific | Cat # A32731 |
| Goat anti- Rabbit Secondary Antibody, Alexa Fluor 555 | Invitrogen/Thermofisher scientific | Cat # A32732 |
| Goat anti- Rabbit Secondary Antibody, Alexa Fluor 647 | Invitrogen/Thermofisher scientific | Cat # A32733 |
| Goat anti- Chicken Secondary Antibody, Alexa Fluor 647 | Invitrogen/Thermofisher scientific | Cat # A32933 |
| Chemicals, peptides, and recombinant proteins | | |
| Sorbitol | Sigma | CAS# 50-70-4 |
| Methyl-β-cyclodextrin | Sigma | CAS# 128446-36-6 |
| γ-cyclodextrin | Sigma | CAS#17465-86-0 |
| N-acetyl-L-hydroxyproline | Sigma | CAS# 2018-61-3 |
| DMSO | Sigma | CAS# 67-68-5 |
| Triton X-100 | Sigma | CAS# 9002-93-1 |
| Glycerol | Sigma | CAS# 56-81-5 |
| Urea | Sigma | CAS# 57-13-6 |
| EdU | Invitrogen/Thermofisher scientific | A10044 |
| Tamoxifen | Sigma | T5648 |
| B27 | GIBCO | 17504044 |
| Nerve growth factor | GIBCO | 13257-019 |
| Hibernate A | Invitrogen/Thermofisher scientific | A1247501 |
| Bacterial and virus strains | | |
| AAV2/9-hEF1a-GFP | Shanghai Taitool Bioscience Co. Ltd | https://www.taitool.com/ |
| AAV2/9-hSYN-GFP | Shanghai Taitool Bioscience Co. Ltd | https://www.taitool.com/ |
| AAV2/PHP.S-hEF1a-DIO-mCherry | Shanghai Taitool Bioscience Co. Ltd | https://www.taitool.com/ |
| AAV2/PHP.S-hSYN-DIO-GFP | Shanghai Taitool Bioscience Co. Ltd | https://www.taitool.com/ |
| AAV2/9-hEF1a-mCherry | Shanghai Taitool Bioscience Co. Ltd | https://www.taitool.com/ |
| AAV2/9-hSYN- mCherry | Shanghai Taitool Bioscience Co. Ltd | https://www.taitool.com/ |
| AAV2/9-CMV-GFP | Shanghai Taitool Bioscience Co. Ltd | https://www.taitool.com/ |
| AAV2/9-CMV- mCherry | Shanghai Taitool Bioscience Co. Ltd | https://www.taitool.com/ |
| Critical commercial assays | | |
| Discover-scTM WTA Kit V2 Discover-scTM WTA Kit V2 | Vazyme Co.,Ltd | N711 |
| Click-iT EdU Alexa Fluor 647 Imaging Kit | Invitrogen/Thermofisher scientific | C10357 |
| Experimental models: Organisms/strains | | |
| Sprague-Dawley (SD) Rat line | Laboratory animal center of Nantong University | https://lac.ntu.edu.cn/ |
| Nestin-CreER^T2^ Rat line | Beijing Biocytogen Co., Ltd. | www.biocytogen.com.cn/ |
| Software and algorithms | | |
| SLM | GENEDENOVO Co., Ltd | https://www.genedenovo.com/ |
| t-SNE | GENEDENOVO Co., Ltd | https://www.genedenovo.com/ |
| ImageJ |  | https://imagej.nih.gov/ij/ |
